# Membranous nephropathy: a retrospective observational study of membranous nephropathy in north east and central London

**DOI:** 10.1186/s12882-017-0615-5

**Published:** 2017-06-21

**Authors:** Sanjana Gupta, John Connolly, Ruth J Pepper, Stephen B Walsh, Magdi M Yaqoob, Robert Kleta, Neil Ashman

**Affiliations:** 1 UCL Centre for Nephrology, 1st Floor, Room 1.7007, Rowland Hill Street, London, NW3 2PF UK; 2 Renal Unit, Barts Health NHS Trust, Whitechapel, London, E1 1BB UK

**Keywords:** Membranous nephropathy, Renal failure, Ethnic differences, Nephrotic syndrome, Risk factors

## Abstract

**Background:**

Membranous nephropathy (MN) is the leading cause of nephrotic syndrome in adults. MN is a clinically heterogeneous disease and it is difficult to accurately predict outcomes (including end stage renal failure) at presentation and whom to treat with potentially toxic therapies. We aimed to identify factors predicting outcome in MN in our cohort from two large tertiary London units by undertaking a retrospective data analysis of 148 biopsy-proven MN patients from North East and Central London between 1995 and 2015.

**Methods:**

Review of clinical and biochemistry databases.

**Results:**

Surprisingly, patients that reached end stage renal failure (ESRF) had a less severe nephrosis compared to those that did not develop ESRF; serum albumin 33 g/L (3.3 g/dL) versus 24 g/L (2.4 g/dL), *p* = 0.002 and urinary protein creatinine ratio (uPCR) 550 mg/mmol (5500 mg/g) versus 902 mg/mmol (9020 mg/g), *p* = 0.0124. The correlation with ESRF was strongest with the presenting creatinine; 215 μmol/L (2.43 mg/dL) compared to 81 μmol/L (0.92 mg/dL), *p* < 0.0001. Patients presenting with creatinine of >120 μmol/L (1.36 mg/dL; corresponding to an eGFR of ≤60 ml/min in non-Black males) had an increased rate of ESRF and a faster decline. Other traditional risk factors for progression were not significantly associated with ESRF.

Black patients presented with higher serum creatinine but no statistically significant difference in the estimated glomerular filtration rate, a higher rate of progression to ESRF and had a poorer response to treatment.

**Conclusions:**

This ethnically diverse cohort does not demonstrate the traditional risk profile associated with development of ESRF. Thus, careful consideration of therapeutic options is crucial, as current risk modelling cannot accurately predict the risk of ESRF. Further studies are required to elucidate the role of antibodies and risk genes.

## Background

Idiopathic membranous nephropathy (IMN) is a serious autoimmune renal disease that is the leading cause of adult nephrotic syndrome and can progress to end stage renal failure (ESRF). Secondary forms exist that are attributable to an underlying cause. In all patients with membranous nephropathy (MN) the pathogenesis involves the development of autoantibodies against antigens present on podocytes. Classic autoimmune disorders have a strong female preponderance [1, 2], whereas with MN males are predominantly affected (with a ratio of approximately 3:1). MN has a variable natural history and tends to develop in a stratified way. It demonstrates an approximate ‘rule of thirds’: in untreated patients, spontaneous complete remission of proteinuria occurs in 5-30% at 5 years [3–5], spontaneous partial remission in 25-40% at 5 years [3–5] and progression to ESRF in 41% at 5 years [4, 6]. The risk of progressing to ESRF is increased in those who are older at presentation, have nephrotic range proteinuria and/or decreased glomerular filtration rate (GFR) at presentation; interestingly it is also increased in males [3, 7, 8]. Asian patients appear to have a better prognosis than non-Asians [7].

Immunomodulatory treatment for MN includes cyclophosphamide (CYC), chlorambucil, calcineurin inhibitors (CNI) - such as cyclosporine A and tacrolimus, rituximab, anti-proliferative agents (AP) - such as mycophenolate mofetil and azathioprine - and corticosteroids. These all predispose to opportunistic infections. Alkylating agents, the gold standard treatment recommended by KDIGO [9], increase cancer risk threefold [10]. To lessen exposure to these therapeutic toxins, there has been much interest in predicting MN patients at risk of progression to ESRF. The predictive accuracy of heavy proteinuria is only 30–50%, and risk modeling with multiple clinical variables (still based on data from 1997) yields a disappointing 80% accuracy rate [11].

Published studies describe ethnically homogenous patient cohorts [4, 12, 13] and therefore we were interested to see if there were differences at diagnosis, treatment or response rate within two tertiary renal London units that cover an extensive and ethnically diverse area of North East and Central London. This retrospective study was undertaken to ascertain if there are differences in our patient population, treatment strategies and remission rates compared to those previously reported.

## Methods

### Patient selection

Our study was performed across two tertiary London Renal Units – The Royal Free Hospital and the Royal London Hospital. We identified adult patients with membranous nephropathy (MN) by searching the clinical renal databases at both centres. We excluded patients that did not have MN. Two hundred forty patients were identified with biopsy proven MN. A further 92 patients were excluded from analysis as there was no serial data available for the 2 year period after the diagnosis of MN was made. The remaining 148 patients were included in the study. Of these 148, 121 had IMN, 4 de novo MN in renal transplants and 23 secondary MN. The patients with secondary MN had a range of causes: 14 systemic lupus erythematosus, 1 scleroderma, 6 hepatitides, 1 malignancy and 1 tuberculosis. The study was retrospective so did not need ethical approval as per NHS Health Research Authority regulation.

### Data collection

Data were collected using the renal databases in addition to local clinical pathology databases. The date of the biopsy was considered to be month 0 – (date of diagnosis) and subsequent data collection based thereafter on this date. Serial data was collected at diagnosis, 1, 2, 3, 6, 12, 18 and 24 months. At each time point serum creatinine, albumin, urine protein creatinine ratio, haemoglobin, immunoglobulins and bicarbonate were recorded. Additionally, the use of renin angiotensin system blocker (RASB) or immunosuppression was recorded at each month. Rates of complications, co-morbidities as well as demographic data such as gender, age and ethnicity was collected. Remission status was calculated based on the standard criteria for complete and partial remission [13].

### Analysis of results

A retrospective analysis was then performed and data analysed using Graphpad Prism 6 (Graphpad software, USA). For parametric data, t-tests were used to compare two data sets, and Mann-Whitney tests for non-parametric data. Contingency tables were analysed with Chi square tests and more than three data sets were compared with ANOVA analysis. Prism 6 was used to formulate the graphs.

## Results

### Baseline characteristics

A total of 148 patients were included in this retrospective study. The baseline characteristics of the study population are described in Table 1.

**Table 1:** Table demonstrating baseline characteristics of all patients. Values are given either as median with interquartile range (IQR) or mean with standard deviation (SD) or percentages

Number of cases	148
Gender Ratio (Male: Female) n	90: 58
Median Age	58 (47–71)
Ethnicity (Asian: Black: White: Unknown) %	31: 24: 36: 9
Median Diagnosis Serum creatinine μmol/L (IQR) (mg/dL)	92 (68–183) (1.04, 0.77–2.07)
Median Diagnosis Serum albumin g/L (IQR) (g/dL)	25 (20–31) (2.5, 2–3.1)
Median Diagnosis urine protein creatinine ratio mg/mmol (IQR) (mg/g)	776 (432 – 1172) (7760, 4320–11,720)
Median Diagnosis cholesterol mmol/L (IQR) (mg/dL)	7.5 (5.75–9.25) (290, 222–357)
Median Diagnosis bicarbonate mmol/L (IQR)	25 (23–28)
Mean Diagnosis haemoglobin g/L (SD)	124.8 (21.68)
Co-morbidities: Hypertension / Diabetes / Recurrent UTIs / Malignancy / Mental health issues n	38 / 24 / 4 / 1 / 2
Complications: thrombotic event / treatment related side effect %	13 / 5
Renin angiotensin system blockade medication use %	84

### Differences at diagnosis

**Fig. 1:**
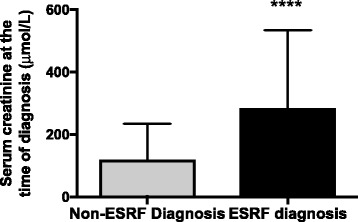
Difference between serum creatinine at presentation between patients ultimately reaching ESRF and non-ESRF

In our study population there were significant differences between characteristics at diagnosis in those that reached ESRF and those that did not. The serum creatinine was significantly higher in those reaching ESRF, 215 μmol/L (124–360) (2.43 mg/dL, 1.4–4) compared to those that did not reach ESRF, 81 μmol/L (64–120) (0.92 mg/dL, 0.72–1.36), *p* < 0.0001; Fig. 1. At each time point reviewed, the difference in the serum creatinine remained statistically significant (month 1, 2, 3, 6, 12, 18 and 24); all *p*-values <0.0001. Serum bicarbonate was lower compared to non-ESRF patients; 21.8 mmol/L ± 0.78 versus 26.0 mmol/L ± 0.45 in non-ESRF patients, *p* < 0.0001. Finally, haemoglobin was also lower in those that reached ESRF; 111.5 g/L ± 2.5 compared to 127.7 g/L ± 2.3, *p* = 0.0001.

**Fig. 2:**
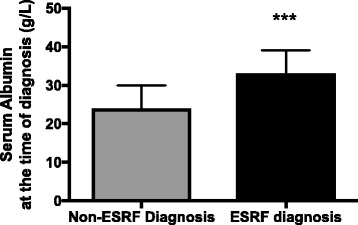
Difference between serum albumin at presentation between patients reaching ESRF and non-ESRF

Patients reaching ESRF had less severe nephrotic syndrome at presentation with a higher serum albumin; median 33 g/L (27–39) (3.3 g/dL) compared to non-ESRF patients; 24 g/L (19–30) (2.4 g/dL), *p* = 0.0002 (Fig. 2). The uPCR was also lower in ESRF patients; 550 mg/mmol (213–985) (5500 mg/g, 2130–9850) compared to 902 mg/mmol (532–1314) (9020 mg/g, 5320–13,140), *p* = 0.0124. The serum cholesterol was lower in patients with ESRF and less severe nephrotic syndrome; 5.7 mmol/L (4.4–7.9) (220 mg/dL, 170–305) compared to non-ESRF patients 7.9 mmol/L (6–9.8) (305 mg/dL, 232–378), *p* = 0.008.

There was no significant difference in gender distribution between the ESRF and non-ESRF groups, the proportion of men reaching ESRF was 27% compared to 22% women, in contrast to 73% of men being non-ESRF and 78% women, *p* = 0.56. There was also no difference in the mean age at presentation (58 ± 1.7 in the non-ESRF group compared to the ESRF group 59 ± 2.4, *p* = 0.67).

Multivariate analysis with a 2-way ANOVA and Bonferroni correction demonstrated that only two significant variables were associated with developing ESRF; the diagnosis serum creatinine and uPCR. Serum creatinine was higher in those reaching ESRF with lower uPCR, *p*-values 0.0134 and <0.0001 respectively.

### Progression of biochemical parameters

Detailed biochemistry was analysed for the 2 years following biopsy diagnosis in all patients. Over this period there was no significant change in the serum creatinine in the non-ESRF patients. There was also no change in the serum bicarbonate or haemoglobin. There was a significant reduction in the cholesterol over the follow up period with treatment; 7.8 mmol/L (301 mg/dL) at admission compared to 4.8 mmol/L (185 mg/dL) at 2 years, *p* < 0.0001. The albumin significantly incremented up to 41 g/L (4.1 g/dL) compared to 24 g/L (2.4 g/dL) at diagnosis, *p* < 0.0001. This mirrored a reduction in uPCR; 903 mg/mmol (9030 mg/g) at diagnosis compared to 119 mg/mmol (1190 mg/g), *p* < 0.0001.

**Fig. 3:**
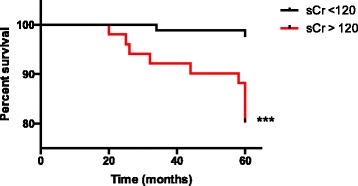
Survival graph showing time to renal failure and difference between presentation creatinine and survival at 5 years

Our patient cohort has a median follow up of 84 months, (longest 211 months / 17.5 years). We therefore examined long-term data on those reaching ESRF. A serum cut off of <120 μmol/L (1.36 mg/dL) was used as this represents an estimated GFR (eGFR) of 60 ml/min/1.73m^2^ (using the abbreviated MDRD formula) in a 50 year old non-Black male. Of patients presenting with a creatinine of <120 μmol/L (1.36 mg/dL) only 10% (9 of 89) reached ESRF within a median time to ESRF of 178 months (under 15 years). Patients with a creatinine >120 μmol/L (1.36 mg/dL) however had an increased rate of developing ESRF; 29 out of 54 (54%) and at a quicker rate with a median time period of 117 months (under 10 years), this difference is statistically significant, *p* < 0.0001, Fig. 3.

### Ethnicity differences

**Fig. 4:**
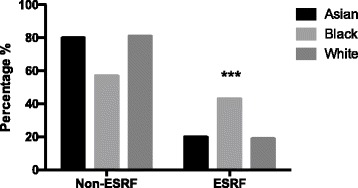
Difference in ethnicities and resultant ESRF rates

Our study population are ethnically diverse, enabling direct comparisons between different ethnic groups. There is an approximate equal spread over the different ethnic groups; Table 2. There was no significant difference in the age at diagnosis for these different groups (mean age – Asian 57, Black 57, White 60). Median creatinine was significantly higher in Black patients (103 μmol/L / 1.17 mg/dL) compared to Asian (69.5 μmol/L / 0.79 mg/dL) and White (87.5 μmol/L / 0.99 mg/dL), ANOVA *p* = 0.0443. However, there was no statistically significant difference between MDRD eGFR though there was a trend to lower eGFR in Black patients. Black patients eGFR was 67 ml/min/1.73m^2^, White patients 71 ml/min/1.73m^2^ and Asian patients the highest at 86 ml/min/1.73m^2^. Despite this, Black patients were more likely to reach ESRF (43%) compared to Asian (20%) and White (19%) patients, Chi-square *p* < 0.0001, see Fig. 4. White patients have higher complete remission rates at 1 year (19%) compared to Asian patients (6.6%) *p* = 0.029, and Black patients (11%) *p* = 0.059.

**Table 2:** Ethnic diversity, number of patients and percentages of different ethnicities within our cohort

Ethnic group	N (%)
Asian	45 (31)
Black	35 (24)
White	54 (36)
Subgroups	
African	14
Caribbean	21
Bangladeshi	9
Chinese	5
Indian	17
Pakistani	9
Middle Eastern	5

### Immunosuppression & Remission Status

One hundred patients were treated with immunosuppression and 48 were treated conservatively. The different therapeutic options and usage rates are summarised in Table 3. All treatments were accompanied by steroids in the form of either oral prednisolone or intravenous methylprednisolone. Those receiving immunosuppression were younger, compared to those treated conservatively (median age of 55 vs. 66 yrs. old, *p* = 0.0016). There was no difference in the creatinine, albumin or uPCR at diagnosis between those that were treated either conservatively or immunosuppressed. Additionally, there was no difference in these three parameters at 1 year between these two groups.

**Table 3:** Different therapeutic options used in our cohort

Treatment	N (%)
Conservative	48 (32)
Cyclophosphamide	36 (24)
Calcineurin inhibitors	38 (26)
Antiproliferative agents (MMF/azathioprine)	21 (14)
Rituximab/steroid monotherapy/other	1/3/1 (Total 3%)

Rates of complete remission were highest with CYC at 25%, the least effective immunosuppressants to achieve complete remission were CNIs at 16%. This contrasts to no immunosuppression with a complete remission rate of 6% and AP agents at 24%. The partial remission rate was better with immunosuppression rather than conservative treatment 29% (CYC 38%, CNI 37%, AP 33%). The lowest rate of no remission was in the CYC group at 36% compared to CNI 47%, AP 43% and conservative management 54%. This suggests superiority of cyclophosphamide in our patient cohort, however the results did not reach statistical significance. Black patients were less likely to be treated with CNI (18%) and more likely to be treated with CYC (31%).

Patients undergoing complete remission were younger (mean age 55) compared to both partial and non-responders (61 and 59 respectively). Responders to treatment, irrespective of treatment strategy, had a lower creatinine at presentation; median 87 μmol/L (0.98 mg/dL) compared to 120 μmol/L (1.36 mg/dL) in the non-responders, *p* = 0.0116. Additionally, responders had lower serum albumin at diagnosis compared to the non-responders (albumin 25.5 ± 0.9 gl/L (2.6 g/dL) compared to 28.5 ± 1.2 g/L (2.9 g/dL), *p* = 0.0476), but there was no statistically significant difference in the uPCR.

## Discussion

This study corroborates findings from previous studies that conclude patients presenting with impaired renal function are more likely to reach ESRF in MN. [14]. However, at odds with these studies, we have shown that our ESRF patients actually present less nephrotic than the non-ESRF patients. The traditional paradigm is that the worse the proteinuria, the worse the risk of ESRF; patients are even risk stratified for treatment based on the degree of proteinuria in MN [7, 12, 15]. In our cohort, patients who were less nephrotic but with worse renal function progressed to ESRF. This may reflect the reduced glomerular filtration rate attenuating proteinuria, resulting in less severe nephrosis [16].

The other known risk factors for ESRF in MN are gender and age [14]; neither were statistically significant in our group. Further, high lipid levels have been found to contribute to glomerulosclerosis and therefore ESRF independent to the severity of nephrosis [17]; however, we found that patients progressing to ESRF had lower serum cholesterol concentrations, emphasising the attenuated nephrosis in the progression group.

Our data supports the main predictor for progression to ESRF in MN being the serum creatinine at diagnosis. In our cohort, at least, it appears that some of the traditional known risk factors for the development of ESRF with MN are less reliable than previously reported. This is not a trivial matter, as strategies to give toxic treatments for MN are currently based on these risk factors.

A significant limitation to our study is the lack of anti-phospholipase A2 receptor antibody status. This was due to the retrospective nature of the study and the lack of historical serum samples, we are now in a process of collecting anti-PLA2R antibody status of all patients in our tertiary MN clinics. Antibody positivity and titre are important as these are associated with severity and outcome of disease [18, 19].

### Ethnic differences

This ethnically diverse group of patients revealed some interesting data. Where details of ethnicity were made available, MN has been reported in homogenous ethnic groups [4, 12, 13]. We found Black patients had worse serum creatinine and lower eGFR (though the eGFR difference was not statistically significant), were more likely to progress to ESRF and were treated more often with cyclophosphamide. There are no comparable studies or reports in Black patients with MN, however, these findings are similar to studies in other renal diseases. It is known that age, sex, race and body weight affect serum creatinine concentration, some of this difference may be due to higher baseline serum creatinine levels found in Black patients and this explains why eGFR differences were not statistically significant [20]. Black patients with lupus nephritis have deteriorating renal function and reduced survival compared to other ethnic groups [21]. Black patients with an eGFR >60 ml/min have a faster rate of decline in renal function irrespective of their albuminuria compared with White patients [22]. The rate of decline persists despite correction of traditional risk factors such as albuminuria, diabetes and hypertension, which suggests an underlying genetic mechanism [22, 23]. There are no studies of MN outcomes in different ethnic groups, however a recent study reviewed the distribution of glomerulopathies in a Southern Californian population. Overall they had lower rates of Black (18.6%) and Asian (8.8%) patients and a larger proportion of Hispanic patients compared to our cohort [24]. There are some reports that Black patients do not respond to CYC as well as other AP agents [21].

There are differences in socioeconomic and biological factors that may explain the faster rate of decline to ESRF in Black patients. Important proposed mechanisms are an interaction of sociodemographic factors with genetic factors such as lower socioeconomic status, chronic stress, psychosocial factors, environmental pollution and differences in access to health care [22]. It should be noted that, like many other studies, we grouped ethnically discrete groups of patients together. For instance, the Asian group included both Indo-Asian and East-Asian patients and the Black group included African and Caribbean patients; these populations are, of course, genetically diverse.

Knowledge about MN has changed significantly as have treatment strategies [4, 12, 13] since the start of the study period. For future studies of MN patients, antibody status and tissue immunohistochemistry of immune deposits and markers of chronic damage correlates are warranted. Furthermore, genomic data would offer insights into the links between ethnicity, gender and outcomes.

## Conclusions

This ethnically diverse cohort does not demonstrate the traditional risk profile associated with development of ESRF. Those responding to treatment have more severe nephrotic syndrome, whereas those reaching ESRF have the worst renal function and lowest proteinuria at diagnosis. There are ethnic differences with Black patients presenting with a trend to lower eGFR and having an increased risk of ESRF. This study highlights the importance of careful consideration of therapeutic options, as current risk modelling cannot accurately predict the risk of ESRF. Further studies are required to elucidate the role of antibodies and risk genes.

## Abbreviations

ANOVA: Analysis of variance
AP: Anti-proliferative agents
CNI: Calcineurin inhibitors
CYC: Cyclophosphamide
ESRF: End stage renal failure
GFR: Glomerular filtration rate
IQR: Interquartile range
KDIGO: Kidney disease improving global outcomes
MN: Membranous nephropathy
RASB: Renin angiotensin system blocker
SD: Standard deviation
uPCR: Urinary protein creatinine ratio
USA: United States of America
